# Intestinal epithelial cell caveolin 1 regulates fatty acid and lipoprotein cholesterol plasma levels

**DOI:** 10.1242/dmm.027300

**Published:** 2017-03-01

**Authors:** Jessica P. Otis, Meng-Chieh Shen, Vanessa Quinlivan, Jennifer L. Anderson, Steven A. Farber

**Affiliations:** 1 Carnegie Institution for Science, Department of Embryology, Baltimore, MD 21218, USA; 2 Johns Hopkins University, Department of Biology, Baltimore, MD 21218, USA

**Keywords:** Caveolin 1, Caveolae, Zebrafish, Knockout mice, Free fatty acids, LDL cholesterol

## Abstract

Caveolae and their structural protein caveolin 1 (CAV1) have roles in cellular lipid processing and systemic lipid metabolism. Global deletion of CAV1 in mice results in insulin resistance and increases in atherogenic plasma lipids and cholesterol, but protects from diet-induced obesity and atherosclerosis. Despite the fundamental role of the intestinal epithelia in the regulation of dietary lipid processing and metabolism, the contributions of CAV1 to lipid metabolism in this tissue have never been directly investigated. In this study the cellular dynamics of intestinal Cav1 were visualized in zebrafish and the metabolic contributions of CAV1 were determined with mice lacking CAV1 in intestinal epithelial cells (CAV1^IEC-KO^). Live imaging of Cav1–GFP and fluorescently labeled caveolae cargos shows localization to the basolateral and lateral enterocyte plasma membrane (PM), suggesting Cav1 mediates transport between enterocytes and the submucosa. CAV1^IEC-KO^ mice are protected from the elevation in circulating fasted low-density lipoprotein (LDL) cholesterol associated with a high-fat diet (HFD), but have increased postprandial LDL cholesterol, total free fatty acids (FFAs), palmitoleic acid, and palmitic acid. The increase in circulating FAs in HFD CAV1^IEC-KO^ mice is mirrored by decreased hepatic FAs, suggesting a non-cell-autonomous role for intestinal epithelial cell CAV1 in promoting hepatic FA storage. In conclusion, CAV1 regulates circulating LDL cholesterol and several FA species via the basolateral PM of enterocytes. These results point to intestinal epithelial cell CAV1 as a potential therapeutic target to lower circulating FFAs and LDL cholesterol, as high levels are associated with development of type II diabetes and cardiovascular disease.

## Abstract (precis)

**Summary:** Caveolin 1, which forms caveolae, localizes to the basolateral membrane of zebrafish intestinal epithelial cells and regulates circulating murine fatty acid and lipoprotein cholesterol levels.

## INTRODUCTION

Caveolae are flask-shaped pits, 50 to 100 nm in diameter, which form in lipid-rich plasma membrane (PM) regions of most vertebrate cells (Parton and Simons, 2007). Caveolar vesicle structure is formed by oligomers of caveolin proteins; ∼144 caveolin proteins are present in a single caveola (Parton and Simons, 2007). CAV1 is synthesized in the endoplasmic reticulum, transported to the Golgi, and upon exit, oligomerizes and associates with lipid-rich membrane regions (Parton and Simons, 2007). A threshold level of membrane cholesterol is required for caveolae to form (Rothberg et al., 1992) and CAV1 can directly bind cholesterol (Murata et al., 1995) and fatty acids (FAs) (Trigatti et al., 1999).

Historically recognized for their endocytic function, caveolae also regulate cell-signaling pathways, internalization of cell-surface receptors and ligands, cell adhesion molecule expression, exocytosis, and transcytosis of caveolae cargos (Parton and Simons, 2007). Caveolae are also emerging players in lipid metabolism. Global CAV1 knockout mice (CAV1^KO^) mice have severe alterations in circulating lipids, including decreased fasting free FAs (FFAs), increased postprandial FFAs (Razani et al., 2002), increased triglycerides (TGs) (Frank et al., 2008; Razani et al., 2002), and increased non-high-density lipoprotein (HDL) total, free and esterified cholesterol (Frank et al., 2008; Heimerl et al., 2008; Razani et al., 2002; Valasek et al., 2005). CAV1^KO^ mice are insulin-resistant (Cohen et al., 2003), but are protected from diet-induced obesity (Razani et al., 2002) and atherosclerosis (Fernandez-Hernando et al., 2009; Frank et al., 2004). The plasma lipid and body mass changes in CAV1^KO^ mice have been proposed to result from a variety of mechanisms including altered lipid droplet architecture, reduced adipocyte lipid droplet formation (Cohen et al., 2004), and impaired adipocyte metabolic flexibility (Asterholm et al., 2012), whereas protection from atherosclerosis is likely a result of decreased endothelial adhesion molecule expression.

The cells that line the intestinal epithelia, enterocytes, are highly specialized to bidirectionally absorb, transport and export large quantities of luminal contents and basolateral plasma components. However, the mechanisms by which dietary lipids are internalized, transported and externalized by enterocytes, as well as how enterocytes receive adequate lipids from adipose stores during fasting, remain incompletely understood. The close association of CAV1 with cholesterol, FAs, lipid droplets and lipid-rich PM regions suggests a role for CAV1 and caveolae in intestinal lipid metabolism (Parton and del Pozo, 2013). CAV1 is expressed, and caveolae form, in the enterocytes of several species including humans and mice (Badizadegan et al., 2000; Field et al., 1998; Marchiando et al., 2010; McConnell et al., 2011; Morroni et al., 2007; Nabeyama and Leblond, 1974; Siddiqi et al., 2013). Although it is known that CAV1 and caveolae are present in enterocytes, several basic aspects of their biology, including subcellular localization and metabolic functions, remain unclear.

Although intestinal cholesterol absorption is not disrupted in mice lacking CAV1 (Valasek et al., 2005), isolated intestinal caveolae contain dietary FAs (Siddiqi et al., 2013) and intestinal lipoprotein cholesterol export influences plasma cholesterol levels, so intestinal CAV1 might significantly impact cholesterol metabolism. Cell culture studies have suggested that CAV1 PM localization might be asymmetric in polarized cells such as intestinal enterocytes; however, reports are conflicting. For example, in human intestinal cells CAV1 localizes asymmetrically, but the pattern varies by cell type: in human T84 colonic adenocarcinoma cells, CAV1 was observed only on lateral membranes (Nusrat et al., 2000); in human intestinal biopsies, CAV1 was found only on the basolateral surface (Badizadegan et al., 2000); and in Caco2 cells, CAV1 localizes to the apical plasma membrane (Field et al., 1998). This imprecise understanding of enterocyte CAV1 localization hampers understanding of its functions in health and disease in the intestine.

A shortage of studies in live, intact animal models has limited our understanding of the contributions of intestinal CAV1 to enterocyte cell biology and global lipid metabolism. Therefore, in this study we harnessed the genetic tractability and optical clarity of the larval zebrafish (*Danio rerio*) to perform live imaging of intestinal Cav1 and caveolae-mediated endocytosis for the first time. The zebrafish digestive system is similar to that of the human, composed of a liver, gallbladder and intestine (Carten and Farber, 2009), and lipid and lipoprotein metabolism are highly conserved (Babin and Vernier, 1989; Otis et al., 2015). Similar to humans and mice, the zebrafish genome contains one *cav1* gene, with two major splice transcripts, and is expressed in the intestine (Nixon et al., 2007). Zebrafish have previously enabled the elucidation of a role for Cav1 in embryonic organogenesis (Fang et al., 2006; Nixon et al., 2007) and live super­-resolution imaging of Cav1 in the embryonic tail (Gabor et al., 2015). Additionally, we employed the power of the mouse model to generate a tissue-specific *Cav1* deletion and determine its contribution to global lipid metabolism. Our transgenic zebrafish and knockout mice, combined with a innovative approach to assay enterocyte endocytosis *in vivo*, allows for an unprecedented understanding of enterocyte CAV1 cell biology, the effects of enterocyte CAV1 on systemic lipid metabolism, and how CAV1 in the intestinal epithelia influence metabolic disease risk through alterations in circulating lipids.

## RESULTS

### Cav1 localizes to the lateral and basolateral PM of zebrafish enterocytes

**Fig. 1.**
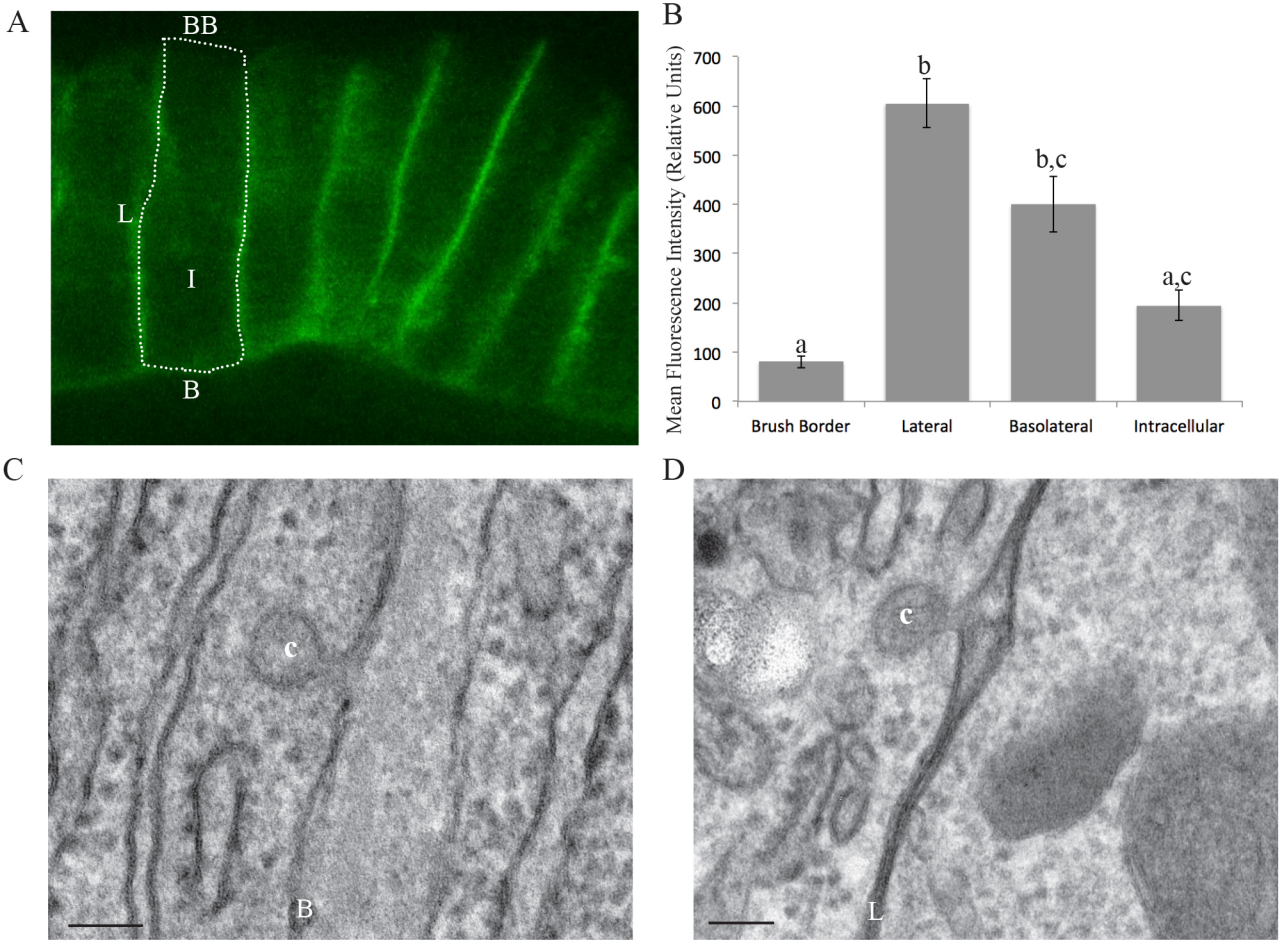
**Cav1 and caveolae localize to the basolateral and lateral PM of enterocytes.** (A) Live imaging of *Tg(hsp70l:cav1-eGFP)* (6 dpf) zebrafish larvae shows localization of Cav1-eGFP to the lateral and basolateral plasma membranes (PM) of enterocytes, but not the luminal brush border. One enterocyte is outlined. (B) Mean fluorescence intensity, in relative units, of Cav1-eGFP in subcellular regions of larval enterocytes. Data is mean±s.e.m, *n*=3: nine fish per replicate, three areas of each region per fish; groups with different letters are significantly different (one-way ANOVA, *P*<0.05). (C,D) Representative EM images of caveola vesicles observed on the basolateral and lateral PMs of larval (6 dpf) (C) and adult (D) zebrafish enterocytes. BB, brush border; L, lateral membrane; B, basolateral membrane; I, intracellular; c, caveolae. Scale bars: 100 nm.

We generated *Tg(hsp70l:cav1-eGFP)* zebrafish that express zebrafish Cav1 tagged with GFP and performed live imaging. Cav1 localizes to the basolateral and lateral PM of intestinal epithelial cells, but is excluded from the luminal brush border (Fig. 1A). Mean fluorescence intensity is 7.5-fold greater on the lateral PM (605.5 relative units) than the brush border (80.7 relative units) (one-way ANOVA, *P*<0.05; Fig. 1B).

As Cav1 protein can exist as either a monomer or an oligomer structuring caveolae, we examined EM sections to determine where caveolae localize. Similar to the localization of Cav1, caveolae are observed on both the basolateral and lateral membranes, but not on the brush border (Fig. 1C,D).

### Caveolae-mediated endocytosis occurs only on the basolateral side of enterocytes

**Fig. 2.**
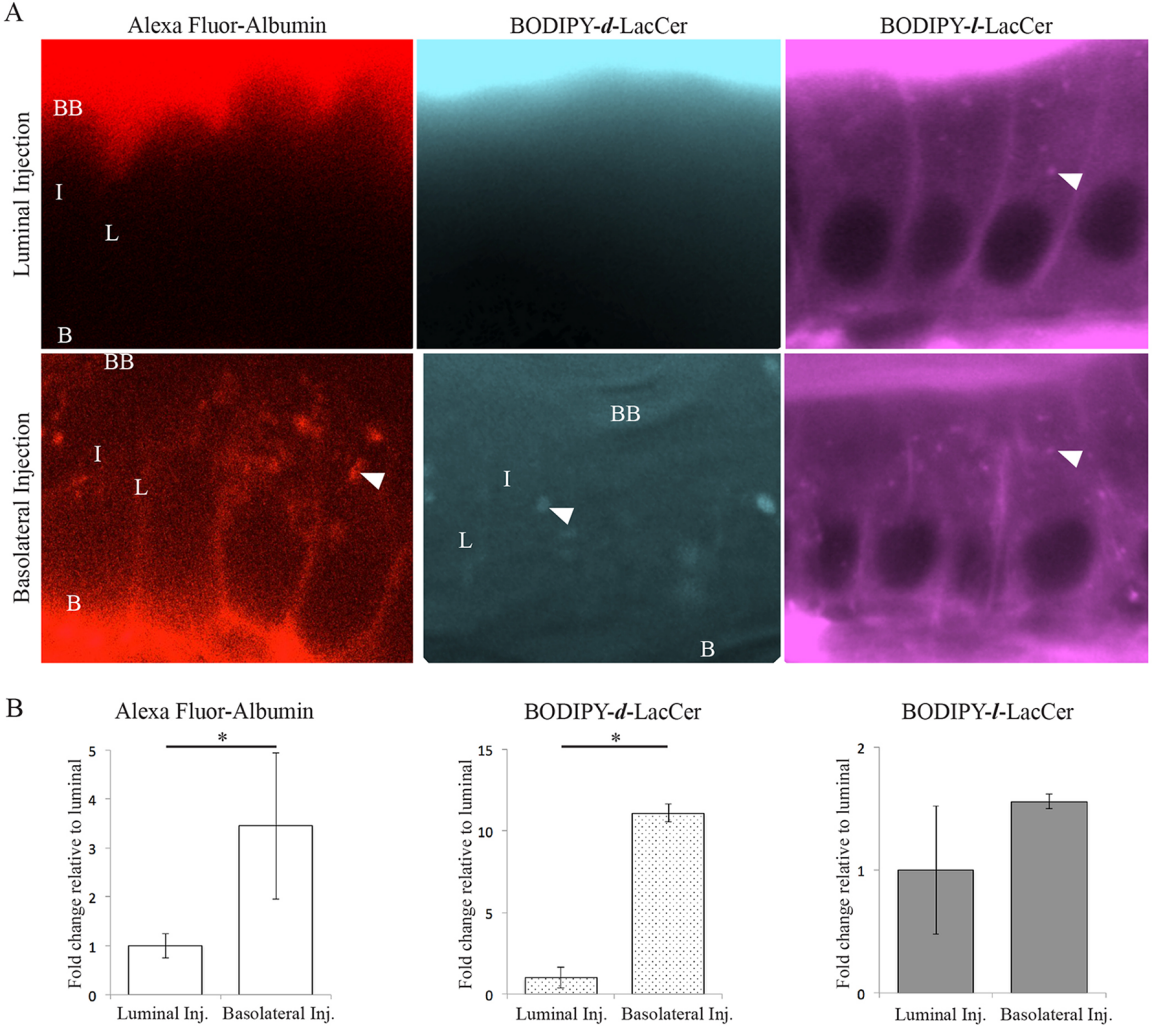
**Fluorescently labeled endocytic cargos enable imaging of caveolar endocytosis in the intact zebrafish intestine.** (A) Representative images show that the caveolar-specific cargos Alexa Fluor–albumin and BODIPY–*d*-LacCer are internalized from the basolateral PM of enterocytes, but not the intestinal lumen. In contrast, the cargo transported specifically by clathrin-coated vesicles, BODIPY–*l*-LacCer, is transported into enterocytes from both the basolateral and luminal PMs. BB, brush border; L, lateral membrane; B, basolateral membrane; I, intracellular; N, nucleus; arrowhead, intracellular puncta. (B) The mean fluorescence intensity of Alexa Fluor–albumin and BODIPY–*d*-LacCer on the lateral PM of enterocytes is significantly greater following basolateral injection compared to luminal injection. In contrast, the mean fluorescence intensity of BODIPY–*l*-LacCer on the lateral PM of enterocytes is the same following basolateral and luminal injection. Data is presented relative to lateral PM mean fluorescence intensity following luminal injection. Mean±s.e.m, *n*=3: nine fish per replicate, three areas of each region per fish; Student's *t*-test; *P<0.05.

The asymmetric PM localization of zebrafish enterocyte Cav1 suggests that caveolae-specific endocytosis occurs between the intestinal epithelia and submucosa, but not the intestinal lumen. To test this hypothesis, we developed a technique to visualize endocytosis in live zebrafish larvae based on cell culture studies that use fluorescently labeled endocytic cargos (Singh et al., 2006). We injected fluorescently labeled endocytic cargos that are internalized specifically by caveolae [Alexa Fluor–albumin (Chanthick et al., 2016; Dobrinskikh et al., 2014; Ghitescu and Bendayan, 1992; Ghitescu et al., 1986; Schubert et al., 2001) and BODIPY–*d*-LacCer (Singh et al., 2003, 2006)] or clathrin-coated vesicles [BODIPY–*l*-LacCer (Singh et al., 2006)] into the basolateral or luminal side of enterocytes. Live confocal imaging indicated if endocytosis took place from the basolateral/lateral PM (basolateral injection) or brush border (luminal injection) in the intestinal epithelia. Whereas BODIPY–*l*-LacCer, the cargo transported specifically by clathrin-coated vesicles, is internalized from both enterocyte PM regions, the caveolae-specific cargos Alexa Fluor–albumin and BODIPY–*d*-LacCer, are only endocytosed from the basolateral PM (Fig. 2A). The fluorescently labeled caveolae-specific cargos localize to similar cellular locations as endogenous Cav1 and Cav1–GFP (lateral and basolateral PM), whereas the clathrin-specific cargo localizes to distinct cellular locations (brush border) (Fig. 2A). The mean fluorescence intensity of the lateral PM was 3.45-fold and 11.1-fold greater following basolateral injection of Alexa Fluor–albumin and BODIPY–*d*-LacCer, respectively, relative to luminal injection (Student's *t*-test, *P*=0.018 and *P*=0.003; Fig. 2B). Two lines of zebrafish with mutations in c*av1* have recently been published (Cao et al., 2016); we hypothesized that uptake of caveolar cargos would be lost in the enterocytes of the fish. Basolateral albumin injections were repeated in the larvae; however, uptake of Alexa Fluor–albumin was not decreased in *cav1^PD1094^*, likely because total *cav1* mRNA is not decreased in this line. This experiment was also attempted in two clutches of *cav1^PD1104^* larvae, and although some fish were viable to adulthood, homozygous mutant larvae did not survive the experimental treatment (data not shown; fish generously provided by Michel Bagnat, Department of Cell Biology, Duke University Medical Center, Durham, NC, USA). These results suggest that caveolae perform a polarized endocytic function in enterocytes, mediating transport between enterocytes and the submucosa, but not the intestinal lumen.

### Generation of intestinal epithelial cell CAV1 knockout mice

**Fig. 3.**
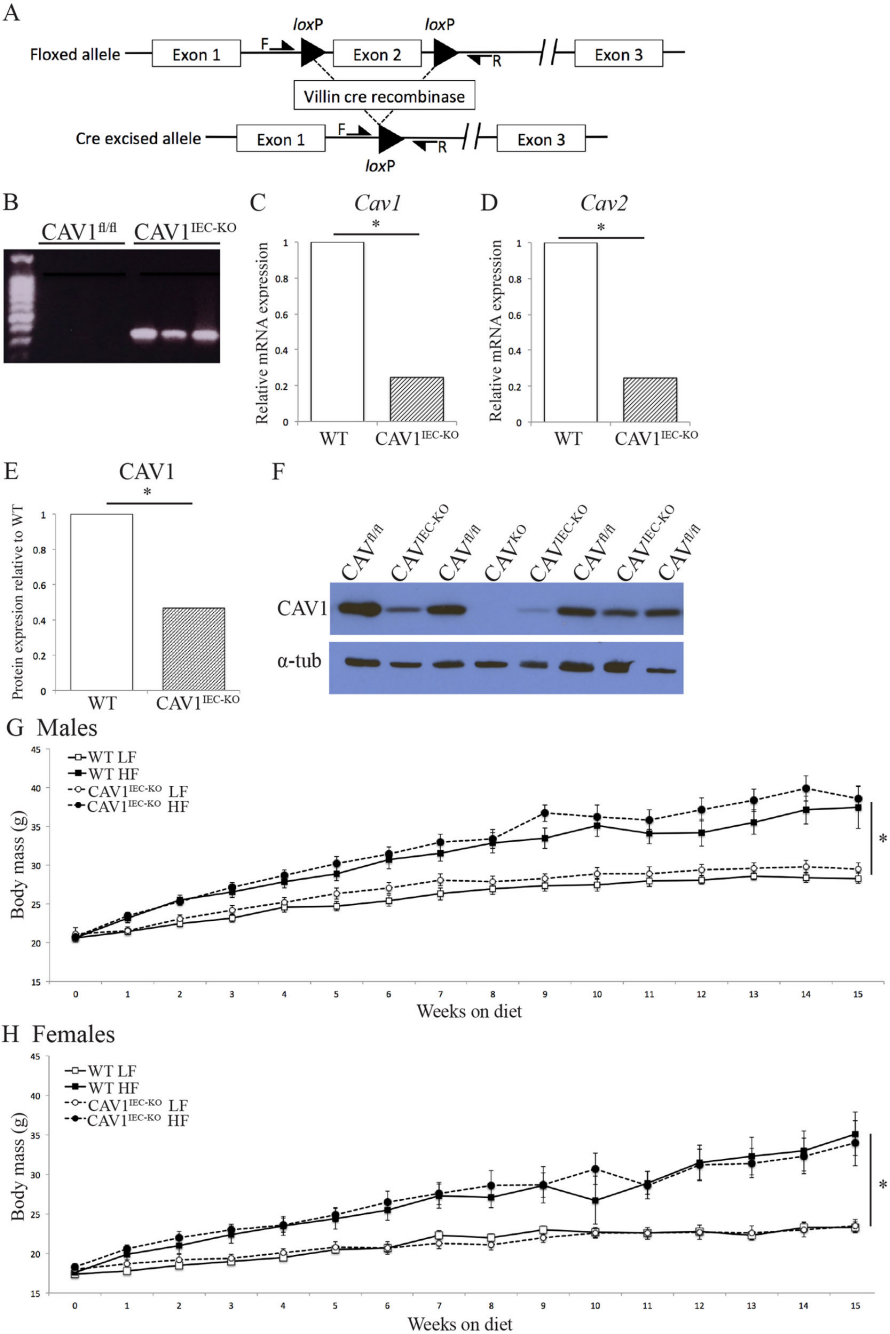
**Deletion of *Cav1* from mouse intestinal epithelial cells (CAV1^IEC-KO^)**. (A) Schematic representation of deletion of *Cav1* in the intestinal epithelium. (B) PCR of genomic DNA from whole mouse jejunum shows that Cre recombination of *Cav1* has occurred in CAV1^IEC-KO^ jejunum but not in *Cav1^fl/fl^* WT littermates. (C) *Cav1* mRNA is decreased 68% in CAV1^IEC-KO^ mouse jejunum as evidenced by RT-PCR (mean, Student's *t*-test, *P=0.01, *n*=10). (D) *Cav2* mRNA is decreased 75% in CAV1^IEC-KO^ mouse jejunum as evidenced by RT-PCR (mean, Student's *t*-test, *P=0.01, *n*=10). (E,F) CAV1 protein is reduced in the jejunum of CAV1^IEC-KO^ mice as measured by western blot (F) and normalized to α-tubulin. (E) Data are expressed relative to *Cav1^fl/fl^* WT CAV1 protein, *n*=3 western blots, five WT and five CAV1^IEC-KO^ mice per blot, Student's *t*-test; *P<0.05. (G,H) Body mass of male (G) and female (H) mice; mice were fed a low-fat (10%) or high-fat (60%) diet starting at 10 weeks (*n*=10-15). HFD mice had significantly higher body mass than LFD mice, but loss of intestinal epithelial cell CAV1 did not affect body mass. Mean±s.e.m, linear regression; *P<0.05.

Although zebrafish are an ideal model to visualize Cav1 and endocytosis, it is extremely challenging to perform tissue-specific gene deletion and measurements of plasma metabolites. To address this challenge, mice lacking CAV1 specifically in intestinal epithelial cells (CAV1^IEC-KO^) were generated by crossing mice with floxed *Cav1* (*Cav1^fl/fl^*) (Cao et al., 2003) with Villin-Cre mice (Madison et al., 2002) (Fig. 3A).

PCR of gDNA from whole jejunum (a mixed tissue sample containing non-genetically modified smooth muscle and vasculature endothelial cells in addition to intestinal epithelial cells lacking CAV1) shows evidence that Cre recombination occurs in the intestine of CAV1^IEC-KO^ mice but not in wild-type (WT) *Cav1^fl/fl^* littermates (Fig. 3B). Real-time PCR of jejunum (tissue includes epithelial cells as well as submucosa and muscle) demonstrates that *Cav1* mRNA is decreased ∼70% in CAV1^IEC-KO^ mice relative to *Cav1^fl/fl^* WT (Student's *t*-test, *P*=0.01; Fig. 3C) and not expressed in the jejunum of negative control CAV1^KO^ mice (data not shown). *Cav2* mRNA is also decreased in CAV1^IEC-KO^ mice, which have 75% less *Cav2* mRNA relative to *Cav1^fl/fl^* WT mice (Student's *t*-test, *P*=0.01; Fig. 3D) (Drab et al., 2001; Razani et al., 2001). Western blot shows CAV1 protein expression is 55% lower in CAV1^IEC-KO^ mouse jejunum than in *Cav1^fl/fl^* WT littermates (Fig. 3E) and that CAV1 is lost in the jejunum of negative control CAV1^KO^ mice (Fig. 3F).

### Loss of intestinal epithelial CAV1 does not affect body mass or glucose metabolism

High-fat diet (HFD) increases the body mass of male and female mice relative to mice fed a low-fat diet (LFD), but CAV1^IEC-KO^ does not protect against diet-induced obesity (16 weeks on diet, 22 weeks old, linear regression; males: *F*=37.7689, *P*<0.0001 for diet; females: *F*=33.8792, *P*<0.0001 for diet; Fig. 3G,H). Fasting plasma glucose (Fig. S1A), glucose tolerance (Fig. S1B,C) and insulin tolerance (Fig. S1D) are similarly unaffected by genotype. Thus, neither body mass nor glucose metabolism influence the following changes in lipid metabolism.

### Deletion of intestinal epithelial CAV1 alters cholesterol levels

**Fig. 4.**
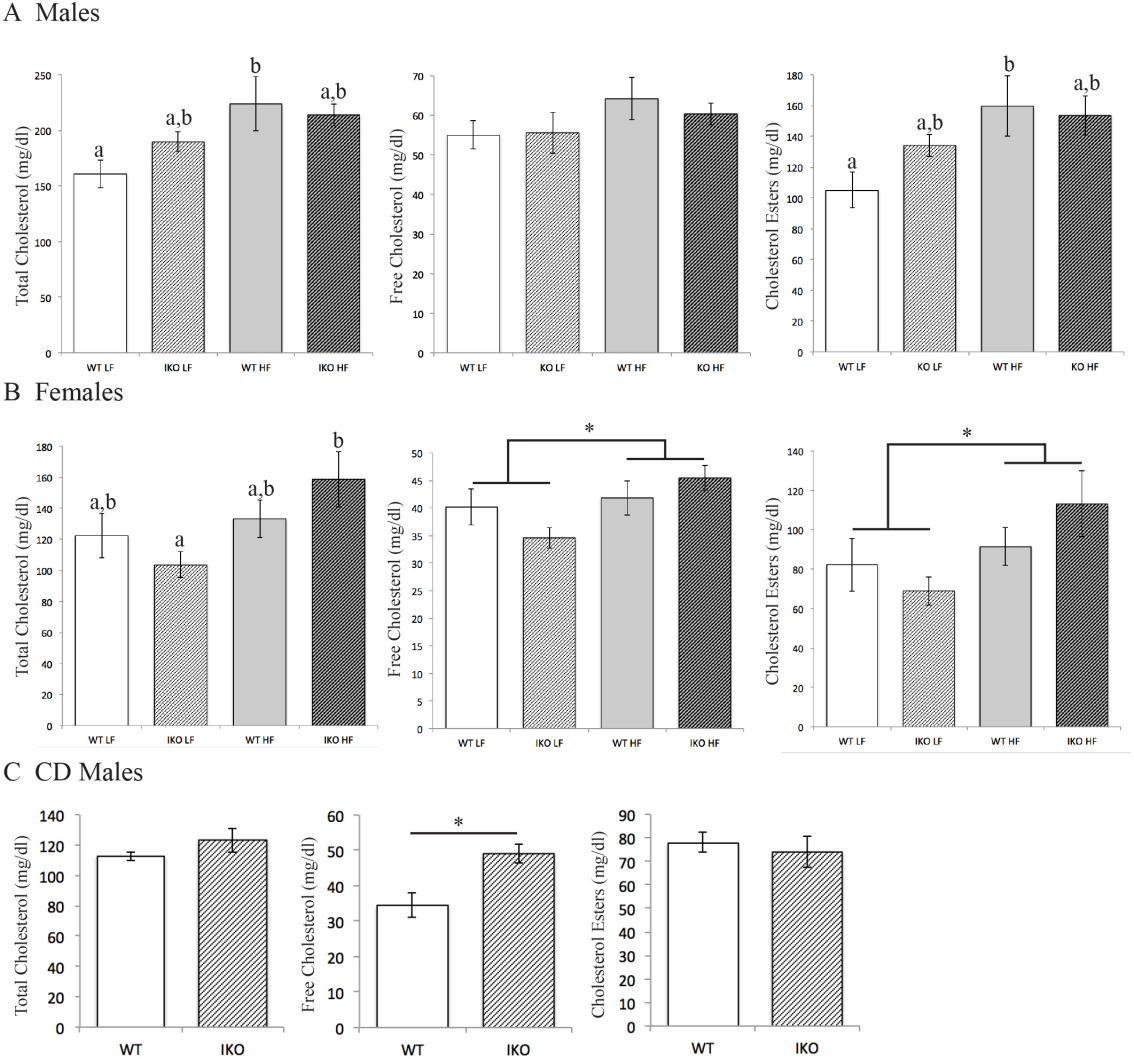
**Loss of CAV1 in the intestinal epithelia alters plasma cholesterol levels.** (A) In male mice, total and esterified plasma cholesterol are elevated by HFD compared with LFD in WT, but not CAV1^IEC-KO^ (IKO), mice following a 4 h fast (*n*=5-8). (B) Conversely, for female mice, total, free, and esterified plasma cholesterol are elevated by HFD compared with LFD in 4 h fasted CAV1^IEC-KO^, but not WT, mice (*n*=8-9). (C) Postprandial male CAV1^IEC-KO^ mice fed CD have greater plasma-free cholesterol mice than WT (*n*=6-8). Data are mean±s.e.m, two-way ANOVA, *P<0.05; groups with different brackets show an effect of diet, groups with different letters are significantly different by post hoc testing.

Deletion of CAV1 in the intestinal epithelia results in changes in fasting and postprandial circulating total and lipoprotein cholesterol, especially when mice were challenged by 16 weeks HFD. Compared with *Cav1^fl/fl^* WTs, male CAV1^IEC-KO^ mice have a greater increase in total and esterified cholesterol upon HFD treatment relative to LFD controls (increase in total cholesterol: 62.9 mg/dl CAV1^IEC-KO^ vs 24.3 mg/dl WT, two-way ANOVA, effect of diet: *F*_1,24_=8.055, *P*=009; increase in esterified cholesterol: 54.4 mg/dl CAV1^IEC-KO^ vs 19.5 mg/dl WT, two-way ANOVA, effect of diet: *F*_1,23_=7.610, *P*=0.01; Fig. 4A). A similar trend for greater total cholesterol is observed in postprandial male mice maintained on chow diet (CD), but only free cholesterol is significantly elevated by these conditions (34.6 mg/dl WT vs 49.1 mg/dl CAV1^IEC-KO^ increase, Student's *t*-test, *P*<0.05; Fig. 4C). It is well established that plasma lipids can show sexual dimorphism, and here, the effects of intestinal CAV1 on plasma cholesterol are sexually dimorphic: *Cav1^fl/fl^* WT females have greater HFD-associated elevations in total cholesterol (10.8 mg/dl CAV1^IEC-KO^ vs 55.1 mg/dl WT, two-way ANOVA, effect of diet: *F*_1,26_=5.881, *P*=0.02) free cholesterol (1.7 mg/dl CAV1^IEC-KO^ vs 10.9 mg/dl WT, two-way ANOVA, effect of diet: *F*_1,26_=5.075, *P*=0.03), and esterified cholesterol compared with CAV1^IEC-KO^ females (9.2 mg/dl CAV1^IEC-KO^ vs 44.3 mg/dl WT, two-way ANOVA, effect of diet: *F*_1,26_=4.707, *P*=0.04; Fig. 4B).

### Intestinal epithelial CAV1 deletion protects against HFD-induced LDL cholesterol increase

**Fig. 5.**
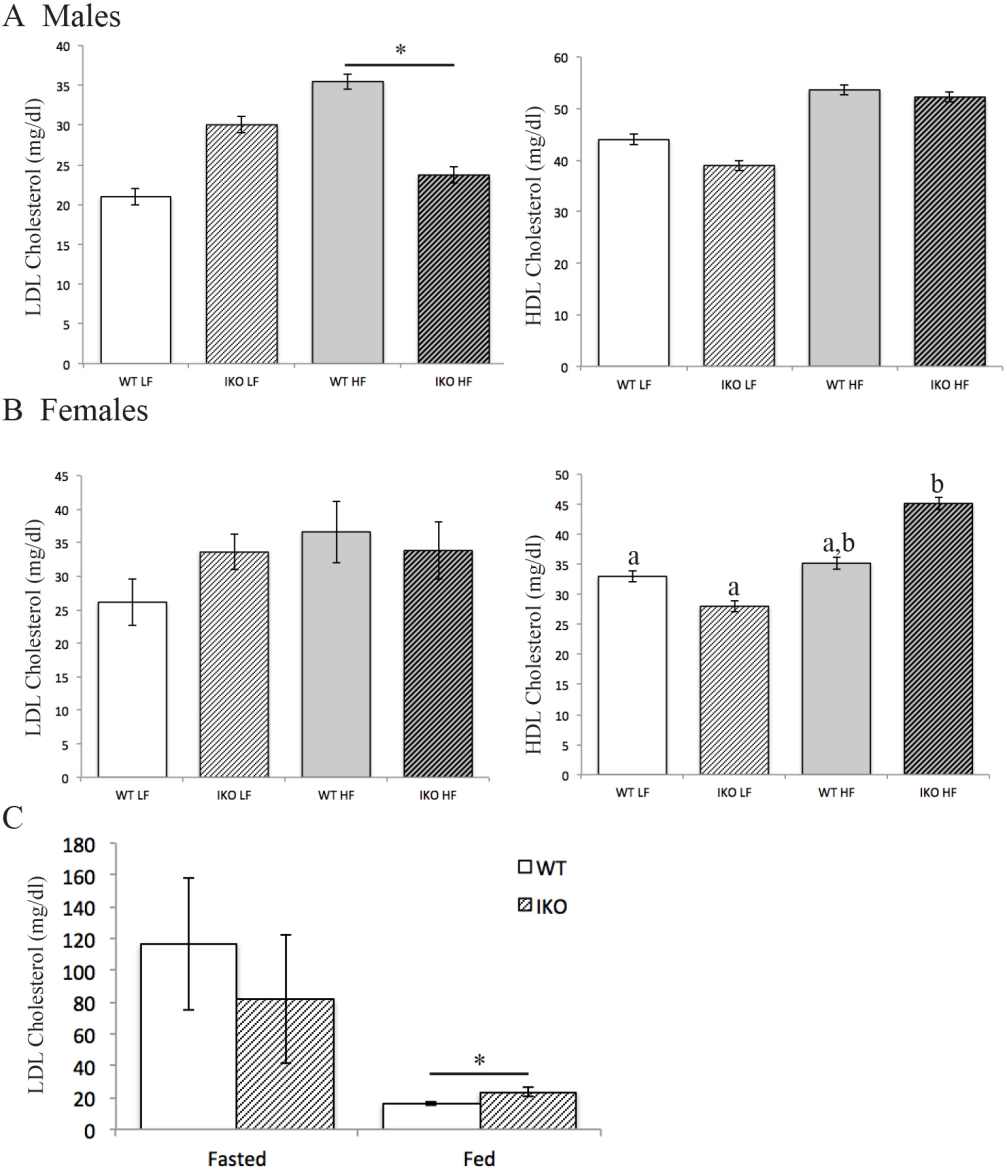
**Lipoprotein cholesterol levels are affected by loss of CAV1 in the intestinal epithelia.** (A,B) Male CAV1^IEC-KO^ (IKO) mice are protected from HFD-induced increase in fasted plasma LDL cholesterol (*n*=6-10, mean±s.e.m, two-way ANOVA, significant interaction between diet and genotype) (A), but female mice are not (*n*=8-9) (B). Female CAV1^IEC-KO^, but not WT, mice have higher fasted plasma HDL cholesterol on HFD than LFD. Mean±s.e.m, two-way ANOVA, groups with different letters are significantly different by post hoc testing. (C) CAV1^IEC-KO^ mice on CD have higher postprandial plasma LDL cholesterol than WT mice. Mean±s.e.m, Student's *t*-test, *P<0.05, *n*=6-9.

Male CAV1^IEC-KO^ mice are protected against the HFD-associated increase in fasted (4 h) plasma low-density lipoprotein cholesterol (LDL-C) seen in *Cav1^fl/fl^* WT littermates (HFD WT: 35.5 mg/dl, 5.5 mg/dl greater than LFD; HFD CAV1^IEC-KO^: 23.7, 6.3 mg/dl less than LFD; two-way ANOVA, significant interaction between diet and genotype, *F*_1,24_=5.261, *P*=0.03; Fig. 5A), though female mice do not exhibit this protection (Fig. 5B). Conversely, postprandial LDL-C is elevated in male CAV1^IEC-KO^ mice (23.4 mg/dl) compared with WT (16.1 mg/dl; Student's *t*-test, *P*<0.05; Fig. 5C). HDL-C is also altered in CAV1^IEC-KO^ mice, as female mice have a greater HFD-induced increase in HDL-C relative to LFD in CAV1^IEC-KO^ mice (13.3 mg/dl for males, 17.1 mg/dl females) compared with WT (9.6 mg/dl for males, 0.2 mg/dl for females; two-way ANOVA, effect of diet: *F*_1,28_=9.516, *P*=0.005, and interaction between diet and genotype *F*_1,28_=5.684, *P*=0.024; Fig. 5A,B).

### Intestinal epithelial CAV1 deletion increases plasma free fatty acids

**Fig. 6.**
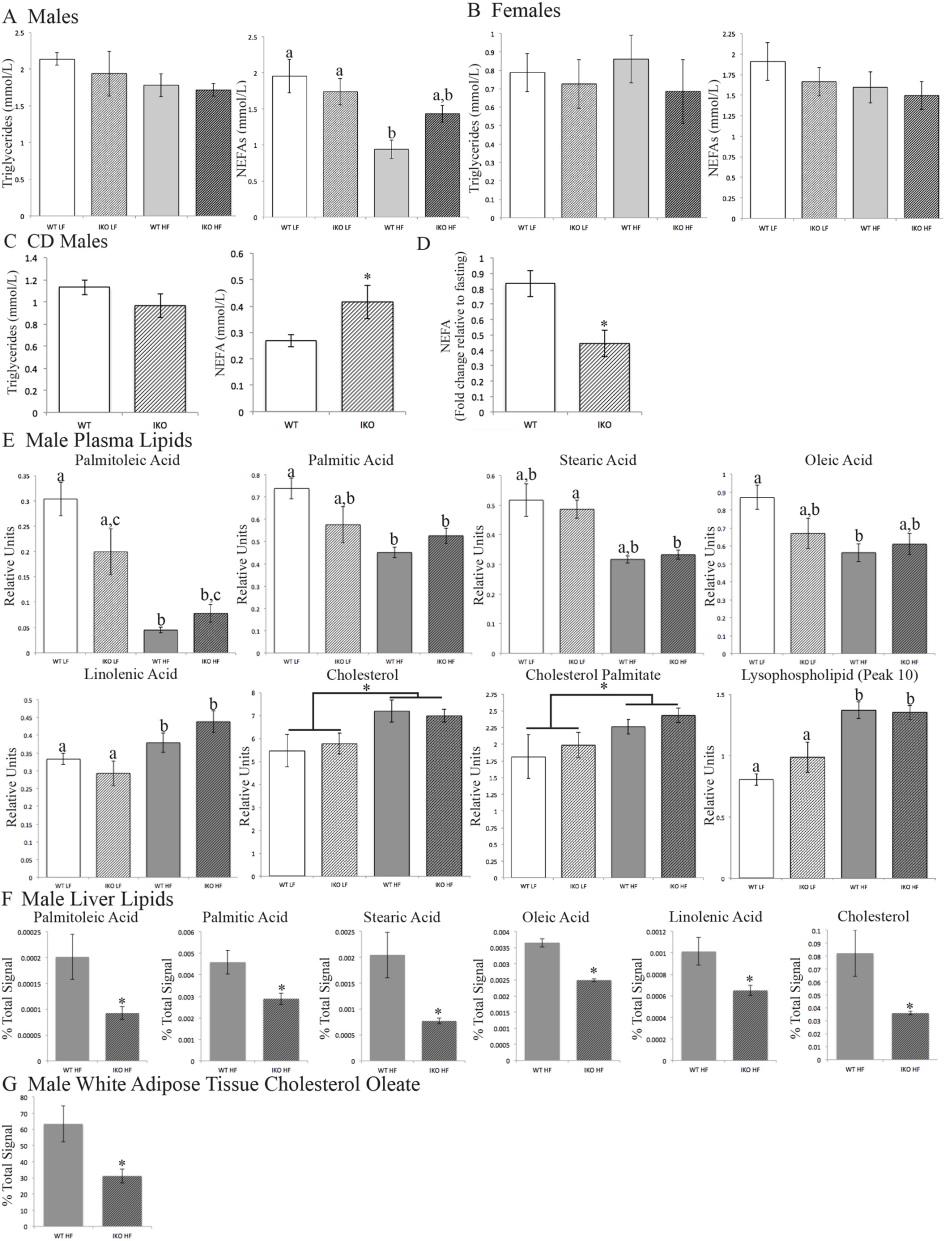
**Intestinal CAV1 deletion alters circulating FFAs but not TGs.** (A) In 4 h fasted male mice, plasma NEFAs are higher in HFD-fed CAV1^IEC-KO^ (IKO) mice than WT mice. Mean±s.e.m, *n*=5-8; two-way ANOVA, means with different letters are significantly different by post hoc testing. (B) Female mice showed no changes in fasted plasma TGs or NEFAs (*n*=8-9). (C,D) NEFAs are also higher in postprandial plasma of male CD CAV1^IEC-KO^ mice compared with WT controls (C) and show a greater fold decrease upon feeding relative to fasting (D). Mean±s.e.m, *n*=6-8; Student's *t*-test, *P<0.05. (E) Lipids measured by HPLC in plasma of male mice fasted 4 h. There are significant effects of diet for all and, for palmitoleic and palmitic acid, the interaction between diet and genotype. Mean±s.e.m, *n*=6; two-way ANOVA, means with different letters are significantly different by post hoc testing, means with different brackets show only a diet effect. (F) Lipids measured by HPLC in liver of male mice fasted 4 h. Mean±s.e.m, *n*=5; Student's *t*-test, *P<0.05. (G) Cholesteryl oleate measured by HPLC in white adipose tissue of male mice fasted 4 h. Mean±s.e.m, *n*=5; Student's *t*-test, *P<0.05.

Loss of intestinal epithelial CAV1 increases plasma non-esterified fatty acids (NEFAs) in fasted male HFD CAV1^IEC-KO^ mice (1.43 mmol/l) compared with HFD *Cav1^fl/fl^* WT littermates (0.94 mmol/l; two-way ANOVA, effect of diet: *F*_1,23_=13.15, *P*=0.0014; Fig. 6A). NEFAs are also greater in postprandial CD-fed male CAV1^IEC-KO^ mice (0.42 mmol/l) than WT mice (0.27 mmol/l; Student's *t*-test, *P*<0.05; Fig. 6C). Moreover, serum NEFAs decrease less in CD-fed male CAV1^IEC-KO^ mice upon feeding (0.44-fold decrease) than WT male mice (0.83-fold decrease; Student's *t*-test, *P*<0.05).

### HPLC lipidomics

To investigate changes in the levels of specific species of plasma NEFAs and cholesteryl esters we performed a lipidomics study. Using HPLC charged aerosol detection (HPLC-CAD), we measured the relative levels of several major plasma lipids including eight FA species, free cholesterol, two cholesteryl esters (cholesteryl oleate and palmitate), and five putative lysophospholipids in the plasma of CAV1^IEC-KO^ and *Cav1^fl/fl^* WT male mice (16 weeks HFD or LFD, 26 weeks old, 4 h fast). Strikingly, palmitoleic acid (16:1) is altered in the same pattern by diet and genotype as total NEFAs (two-way ANOVA, effect of diet: *F*_1,20_=41.70, *P*<0.0001; effect of genotype: *F*_1,20_=5.438, *P*=0.03; Fig. 6E). Similarly, palmitic acid (16:0) is significantly affected by both diet and the interaction between diet and genotype (two-way ANOVA, effect of diet: *F*_1,20_=10.77, *P*=0.0037; interaction effect: *F*_1,20_=5.388, *P*=0.031; Fig. 6E). Stearic acid (18:0) is significantly elevated by HFD compared with LFD in CAV1^IEC-KO^ mice (two-way ANOVA, *F*_1,20_=11.95, *P*=0.0025; Fig. 6E). Oleic acid (18:1) and linolenic acid (18:3) are both higher in LFD than HFD fed mice (diet effect found by two-way ANOVA, oleic acid: *F*_1,20_=7.664, *P*=0.0119; linolenic acid: *F*_1,20_=28.72, *P<*0.0001; Fig. 6E). Neither diet nor genotype affects any of the other FAs measured (22:6, 20:4 and 18:2). In sum, palmitoleic, palmitic, stearic and oleic acid might contribute to the observed elevation of total NEFAs in LFD mice compared with HFD mice.

As described above, cholesterol assay kits found that diet affects total and esterified plasma cholesterol in CAV1^IEC-KO^ mice (Fig. 4). HPLC analysis identified a direct contribution of free cholesterol (two-way ANOVA, effect of diet: *F*_1,20_=8.693, *P*=0.008) and cholesteryl palmitate (two-way ANOVA, effect of diet: *F*_1,20_=4.800, *P*=0.04), which are both increased by HFD (Fig. 6E). Finally, one putative lysophospholipid (peak 10) that could not be identified with standards is also increased by HFD (two-way ANOVA, effect of diet: *F*_1,20_=33.25, *P*<0.0001; Fig. 6E).

To investigate the mechanism underlying the elevation in plasma NEFAs in male HFD CAV1^IEC-KO^ relative to male HFD WT mice, liver and white adipose lipids were also measured by HPLC in male HFD mice. Four of the FAs that were decreased in CAV1^IEC-KO^ plasma – palmitoleic, palmitic, stearic, oleic and linolenic acids – were also significantly decreased in the liver (Fig. 6F). It is striking that all of the FAs that are decreased in the liver of CAV1^IEC-KO^ mice, with the exception of stearic acid, have a trend to be increased in the plasma where total NEFAs are increased. Additionally, hepatic cholesterol is lower in HFD CAV1^IEC-KO^ mice (Fig. 6F). No differences were observed in triglycerides, cholesterol esters, or, similar to plasma, 22:6, 20:4 or 18:2 in the liver of HFD mice (data not shown). Of all these lipids, only cholesteryl oleate varied in the white adipose tissue, showing a decrease in CAV1^IEC-KO^ mice (Fig. 6G; data for other lipids not shown).

## DISCUSSION

The global obesity epidemic has caused an explosion in the prevalence of metabolic diseases such as type II diabetes and cardiovascular disease. Intensive efforts have focused on the identification of therapeutic targets to better prevent and treat metabolic syndrome. Previous work had identified functional roles of adipocyte and endothelial CAV1 in susceptibility to diet-induced obesity (Razani et al., 2002), insulin resistance (Cohen et al., 2003) and atherosclerosis (Frank et al., 2004). In this study, we expanded upon these findings by using zebrafish larvae to visualize the localization of enterocyte Cav1 and caveolar endocytosis, and a knockout mouse model to identify a role for CAV1 in the intestinal epithelia in the regulation of plasma FAs and LDL cholesterol, lipids that contribute to the development of several metabolic diseases.

Although it is known that CAV1 is expressed in the intestinal epithelium (Field et al., 1998), the intracellular localization of this protein has remained ambiguous: lateral PM localization in Caco-2 colon-derived cells (Vogel et al., 1998); either the brush border or lateral and basolateral PM localization in Caco-2 cells depending on the fix, permeabilization method, and antibody used (Field et al., 1998); lateral membrane of T84 colon-derived cells (Badizadegan et al., 2000); cytoplasmic vesicles and lateral PM at adherens and tight junctions in mouse jejunum (Marchiando et al., 2010); low levels on the mouse small intestine brush border (McConnell et al., 2011); cytoplasmic vesicles in mouse colon (Nabeyama and Leblond, 1974); deep apical tubules in pig small intestine (Hansen et al., 2003); human small intestine cytoplasmic vesicles (Morroni et al., 2007); and apical PM and cytoplasmic vesicles in *Caenorhabditis* *elegans* intestine (Parker et al., 2009). Here, our *in vivo* study reveals that Cav1 localizes asymmetrically to lateral and basolateral enterocyte PM in larval zebrafish. CAV1 is a FA- and cholesterol-binding protein and caveolae can only form in lipid-rich PM regions. Although these lipid-rich regions do form on the enterocyte brush border (Hansen et al., 2001), this membrane has a lipid composition distinct from the lateral and basolateral PM, with more glycolipids and less cholesterol and sphigomyelin (Danielsen and Hansen, 2006). It is possible that the relative scarcity of cholesterol in the brush border excludes CAV1 and caveolae from this PM. Unfortunately, the cellular localization of mouse CAV1 in the intestinal epithelia could not be determined because of non-specific antibody binding that we observed in enterocytes.

Fluorescently labeled endocytic cargos are valuable tools to visualize various types of endocytosis in cultured cells (Singh et al., 2006, 2007). Here, we extended this technology to image the enterocyte PM regions that likely perform caveolae-mediated endocytosis *in vivo*. The results suggesting that caveolae-mediated endocytic activity in enterocytes is asymmetric points to a role for caveolae in vesicular transport between enterocytes and the body, but not the intestinal lumen. The optical clarity of larval zebrafish and advances in mouse vital imaging present the opportunity to extend this technique to investigations of caveola- and clathrin-mediated endocytosis in a multitude of tissues in the context of health or disease.

Prior studies have reported increased fasting plasma cholesterol in male CAV1^KO^ mice, largely consisting of elevations in VLDL and LDL cholesterol resulting from adipocyte lipid storage defects and decreased aortic and hepatic LDL cholesterol uptake (Frank et al., 2008; Valasek et al., 2005). Our studies find that the intestinal epithelium does not mediate these changes, as we did not observe differences in plasma cholesterol or cholesterol palmitate in CAV1^IEC-KO^ mice. However, we did find that LDL cholesterol levels were increased postprandially and decreased during fasting (when hepatic cholesterol is also decreased) in male HFD CAV1^IEC-KO^ mice; this is opposite of the pattern observed in CAV1^KO^ mice. Another striking observation is that male CAV1^IEC-KO^ mice have increased fasting plasma NEFAs (HFD), mirrored by decreases in several hepatic FAs, and decreased postprandial NEFAs (CD). The literature regarding the effect of total body CAV1 deletion on circulating FFAs is conflicting, with reports of higher fasting and postprandial FFAs (Asterholm et al., 2012; Razani et al., 2002), decreased fasting FFAs (Cohen et al., 2004), or no changes (Valasek et al., 2005).

First, we hypothesized that the changes in LDL cholesterol and FFAs could be mediated by the cluster of differentiation 36 scavenger receptor (CD36). CD36 delays LDL cholesterol clearance (Luangrath et al., 2008), so impairment of its function in CAV1^IEC-KO^ mice could accelerate LDL cholesterol clearance, causing the observed decrease in fasting LDL cholesterol. Increased postprandial LDL cholesterol could also be explained, as CD36 facilitates cellular cholesterol uptake (Nassir et al., 2007). Additionally, FFAs could be increased due to impaired CD36 localization and function, as CD36 facilitates FA uptake (Nassir et al., 2007; Pepino et al., 2014) and its deletion increases serum FFAs (Febbraio et al., 1999). CD36 is highly expressed in the proximal intestine (Poirier et al., 1996) and CAV1 is necessary for CD36 to properly localize to the PM of mouse embryonic fibroblasts (Ring et al., 2006). Therefore, we speculated that CD36 PM localization and or expression might be disregulated in CAV1^IEC-KO^ mice. However, contrary to the previous findings in mouse embryonic fibroblasts, no change in CD36 localization was observed in the intestinal epithelia of HFD-treated CAV1^IEC-KO^ mice (Fig. S2A) and no decreases in mRNA or protein expression were observed (data not shown). In support of this conclusion, it would be expected that hepatic cholesterol would increase if the CD36-medated LDL cholesterol clearance delay was perturbed, but instead HPLC analysis found a decrease in hepatic cholesterol.

Second, changes in circulating FFAs in CAV1^IEC-KO^ mice could be mediated by changes in intestinal albumin uptake. Circulating FFAs are transported by albumin, as much as 18% of which is absorbed and catabolized by the intestine (Yedgar et al., 1983). Indeed, we showed that Alexa Fluor–albumin is internalized by caveolae on the basolateral PM of enterocytes. If this were true a concomitant increase in plasma albumin would be expected; however, this was not observed (Fig. S2B). As circulating albumin levels are tightly regulated, this hypothesis cannot be ruled out because of a lack of direct measurement of albumin flux from the liver. Nonetheless, in further support of our conclusion, increased postprandial plasma lipids likely do not result from altered intestinal processing because no changes in serum NEFAs or TGs were observed by an oral lipid tolerance test (Fig. S3).

Third, we hypothesized that CAV1^IEC-KO^ mice might have decreased intestinal insulin signaling, and thus insulin stimulated plasma FA uptake, underlying the observed increase in circulating NEFAs. This hypothesis is supported by the fact that insulin signaling is present in the intestine (Veilleux et al., 2014), the insulin receptor localizes to caveolae (Gustavsson et al., 1999), and that global CAV1 knockout mice are insulin resistant (Cohen et al., 2003). Substantiating this hypothesis, insulin receptor mRNA is significantly decreased in the jejunum of CAV1^IEC-KO^ mice compared with controls (Fig. S4), but no change in insulin receptor subunit β protein was observed by western blot (data not shown). Importantly, there might be a non-IEC-autonomous mechanism (insulin signaling or otherwise) by which hepatic FA uptake is decreased or secretion is increased leading to the observed decrease in several hepatic FAs and corresponding increase in circulating NEFAs. Similarly, loss of CAV1 in IEC might underlie a non-cell-autonomous mechanism by which hepatic cholesterol is decreased, limiting the amount of LDL cholesterol that can be secreted, causing the decrease in LDL cholesterol observed in CAV1^IEC-KO^ mice.

HPLC determined that palmitoleic and palmitic acid contribute to the overall increase in fasting NEFAs observed in HFD CAV1^IEC-KO^ mice. Palmitoleic acid is an omega-7 mono-unsaturated FA that is enriched in endothelial caveolae (Gafencu et al., 1998). If palmitoleic acid is also preferentially carried in enterocyte caveolae, it follows that loss of CAV1 could result in decreased palmitoleic acid uptake, and the observed plasma elevation. Further investigation of the detailed mechanism would be of interest as palmitoleic acid is a bioactive lipid; dietary supplementation has plasma-lipid-lowering, anti-diabetic, and anti-inflammatory activity (Cao et al., 2008), but elevated plasma palmitoleic acid is correlated with increased heart failure (Djousse et al., 2012) and non-alcoholic fatty liver disease (Puri et al., 2009). It is possible that no influence of genotype on plasma palmitoleic acid levels in LFD mice was found simply because it comprises a much smaller proportion of the diet (0.04% of total LFD vs. 0.44% of total HFD by mass). Caveolae are also enriched in palmitic acid (Cai et al., 2013) so plasma palmitic acid might in higher in CAV1^IEC-KO^ than WT mice due to the mechanism proposed above. As CAV1 is palmitoylated (Dietzen et al., 1995), decreased activity in this pathway might contribute to the plasma palmitic acid elevation.

Finally, the sexual dimorphism observed in the changes in plasma lipids in CAV1^IEC-KO^ mice must be addressed. It is well established that male and female animals have metabolic differences; in C57BL/6J mice, this includes differences in lipid and FA metabolism gene expression in multiple tissues (Yang et al., 2006). Moreover, sex-specific metabolic differences have been observed previously in global CAV1 KO mice; decreased body mass on chow diet compared with WT in male, but not female, mice and increased food intake relative to WT in female, but not male, mice (Razani et al., 2002). However, neither sex had increased circulating free FAs compared with WT, and LDL cholesterol was only measured in male mice, where it was increased compared with WT (Razani et al., 2002). In contrast to this previous study, we report that CAV1^IEC-KO^ male, but not female, mice were protected from the HFD-induced increase in plasma LDL cholesterol observed in WT. Although the mechanism for this difference is unknown, it is possible that sexual dimorphism in estrogen receptor signaling underlies this difference. When estrogen binds the estrogen receptor α (ERα) transcription factor, ERα activates transcription of the LDL receptor (LDLR), which takes up LDL cholesterol from circulation (Cooper et al., 1987). CAV1 has been shown to potentiate the action of ERα in cultured cells (Schlegel et al., 1999). Additionally, female mice of mixed genetic background (129/Pas×C57BL/6J, ∼90% C57BL/6J) have higher *Ldlr* and *Cd36* hepatic gene expression compared with males (Lorbek et al., 2013). Therefore, because estrogen levels are higher in females, differences in estrogen-stimulated LDLR transcription and subsequent cellular uptake might underlie the observed sex-specific differences in LDL cholesterol in CAV1^IEC-KO^ mice. Furthermore, female CAV1^IEC-KO^ mice had significantly increased HDL cholesterol on HFD compared with LFD; this difference was not observed in WT females, and the trend was non-significant in males. These findings are consistent with Link et al.'s report on the role of sex chromosomes that mice with female (XX) chromosomes have higher HDL cholesterol levels than male (XY) mice (Link et al., 2015). The observed sexual dimorphism does not preclude intestinal epithelial CAV1 as a potential therapeutic target for metabolic disease as many pharmaceuticals currently in use have sexually dimorphic actions, including the LDL cholesterol-lowering drug fenofibrate (Yoon et al., 2002). Nor does the modest decrease in LDL cholesterol preclude intestinal epithelial CAV1 as a therapeutic target, as even a 1% reduction in LDL cholesterol leads to a 1% decrease in cardiovascular disease risk (Baigent et al., 2005).

In conclusion, this study demonstrated that CAV1 localizes to, and caveolar endocytosis occurs on, the lateral and basolateral PM of intestinal enterocytes. Although the detailed cellular mechanism remains to be elucidated, it is clear that CAV1 on enterocyte basolateral membranes influences circulating levels of LDL cholesterol and NEFAs, specifically palmitoleic and palmitic acid. Elevated plasma FAs and LDL cholesterol are associated with metabolic disease, including type II diabetes and cardiovascular disease. The results of this study indicate that CAV1 in the intestinal epithelium might serve as a therapeutic target to lower circulating FFAs and LDL cholesterol and prevent disease in males.

## MATERIALS AND METHODS

### Generation of transgenic zebrafish

Zebrafish research was approved by the Carnegie Institution Department of Embryology IACUC Committee (protocol #139). Zebrafish were housed at 28°C with a 14 h:10 h light:dark cycle. *Tg(hsp70l:cav1-eGFP)* zebrafish were created with the tol2-Gateway system (Kwan et al., 2007). Zebrafish *cav1* was cloned from Image Consortium plasmid #3719638 (fb95c12) and Gateway cloning constructed *hsp70l:cav1-eGFP*: the zebrafish *heat shock cognate 70-kd protein, like* (*hsp70l*) promoter driving *cav1* tagged with eGFP [provided by Chi-bin Chien (Kwan et al., 2007)]. This plasmid was injected with tol2 transposase for genome integration with a microforged glass needle (P-97 Flaming/Brown micropipette puller, Stutter Instruments, Novato, CA) connected to a nitrogen gas pressure injector (PLI 100, Harvard Apparatus, Cambridge, MA) into 1-2-cell zebrafish embryos (AB background). F0 larvae were heat shocked [45 min, 37°C, in 15 ml embryo media (EM)] at 6 days post-fertilization (6 dpf) and screened for mosaic Cav1-eGFP expression 4-6 h later. Three independent stable lines were established and all further experimentation was undertaken in stable transgenic animals.

### Live imaging of zebrafish larvae and quantification of fluorescence

Approximately 24 h prior to imaging, larvae were heat shocked as described above to induce Cav1-eGFP expression. Larvae were anesthetized with tricaine (Sigma-Aldrich, St Louis, MO) and mounted in 3% methyl cellulose (Sigma-Aldrich) under a coverslip (Carten et al., 2011). Live *Tg(hsp70:cav1-eGFP)* larvae were imaged on a SP-2 confocal microscope (Leica Microsytems, Deerfield, IL) with an argon laser under a 63× oil immersion objective. Images were collected as 12 bit and analyzed with Metamorph software (Molecular Devices, Sunnyvale, CA). Regions of the brush border, lateral and basolateral PM, as well as intracellular regions, were outlined for quantification of mean fluorescence intensity (three regions of interest for each cellular area, three images per fish, three fish per experiment, for a total of three experiments or nine fish).

### Electron microscopy and immunofluorescence

Larval (6 dpf) zebrafish and intestines from adult zebrafish and were collected for EM. Prior to euthanasia adult fish were fed on a lipid-rich, hard-boiled chicken egg yolk for 1 h *ad lib*; larvae studied were lecithotrophic (6 dpf) and thus were not provided exogenous food. EM samples were fixed in 3% glutaraldehyde (Electron Microscopy Sciences, Hatfield, PA) and 1% formaldehyde, post-fixed in reduced osmium (Electron Microscopy Sciences), stained with uranyl acetate (Fisher Scientific), embedded in Epon 812 resin (Ladd Research Industries, Williston, VT), and imaged on a Technai-12 electron microscope (FEI, Hillsboro, OR) with a 794 multiscan camera (Gatan, Pleasanton, CA).

Mouse CAV1 IF was attempted on paraffin sections and cryosections of jejunum collected after a 4 h fast, but non-specific fluorescence was observed in enterocytes, even in the negative control, global CAV1 KO mice. The antibodies tested at a range of dilutions were: BD Biosciences/Transduction Labs #610059, 610057 and 610406, Santa Cruz Biotechnology #sc-894, Abcam #ab2910, and Cell Signaling #3238 s. The antigen retrieval methods tested were Tris-EDTA buffer, sodium citrate buffer, and Diva Decloaker (Biocare Medical, Concord, CA).

### Imaging fluorescently labeled endocytic cargos *in vivo*

WT Larvae (6 dpf) were anesthetized in tricaine and mounted in 1.2% low-melt agarose (Sigma-Aldrich) in EM. A microforged glass needle was loaded with 4,4-difluoro-4-bora-3a, 4a-diaza-S-indacene-*l*-threo-lactosylceramide (BODIPY–*l*-LacCer) (provided by David Marks, Thoracic Disease Research Unit, Mayo Clinic and Foundation, Rochester, MN, USA) or BODIPY–*d*-erythro-LacCer (#895279, Invitrogen) at a concentration of 2.5 μg/μl in 30% ethanol and 70% embryo media, or Alexa Fluor 594–albumin (#A13101, Fisher Scientific, Pittsburg, PA) at a concentration of 5 μg/μl in PBS. A nitrogen-pressured injection rig was used to inject 2 nl to the basolateral side of the intestine or into the intestinal lumen. Larvae were freed from the agarose, allowed to recover for 30 min (LacCer) or 1 h (albumin) while swimming freely, re-anesthetized, mounted in 3% methyl cellulose under a coverslip, placed on ice to stop endocytosis (Bourseau-Guilmain et al., 2016), imaged, and the same sample size was analyzed with Metamorph software as described above.

### Breeding CAV1^IEC-KO^ mice

Mouse research was approved by the Carnegie Institution Department of Embryology IACUC Committee (protocol #156). Mice were housed at 20-21°C with a 12 h:12 h light cycle. Mice with a floxed *Cav1* allele (provided by Michael Elliot; Cao et al., 2003) were crossed with Tg(Vil-cre)997Gum/J mice (#004586, Jackson Labs, Bar Harbor, ME), which express Cre recombinase specifically in the intestinal epithelium, producing mice lacking *Cav1* specifically in the intestinal epithelium (CAV1^IEC-KO^, back crossed to C57BL/6 6 times; Fig. 1A). All experiments were performed with CAV1^IEC-KO^ versus *Cav1^fl/fl^* (WT) littermates.

Mice were genotyped for floxed *Cav1* (350 bp band following PCR with F1: TTC TGT GTG CAA GCC TTT CC and R1: GTG TGC GCG TCA TAC ACT TG) and Vil-Cre PCR (1100 bp band following PCR with F: GTG TGG GAC AGA GAA CAA ACC and R: ACA TCT TCA GGT TCT GCG GG). The occurrence of Cre recombination in the intestine of CAV1^IEC-KO^ mice was verified by the presence of a 350 bp band (F1 primer above, R2: GGG GAG GAG TAG AAG GTG GC; Cao et al., 2003) on genomic DNA isolated from whole jejunum segments by NaOH extraction (Truett et al., 2000). *Cav1^tm1Mls^* (CAV1^KO^) mice lacking functional *Cav1* in all tissues were used as controls (#007083, Jackson Labs, Bar Harbor, ME).

### Real-time PCR

*Cav1* and *Cav2* mRNA levels were measured in whole jejunum by RT-PCR. mRNA was isolated with Trizol (Invitrogen), cDNA was synthesized with AMV reverse transcriptase (New England Biolabs, Ipswich, MA), and RT-PCR was run with the PrimePCR SYBR Green assay for mouse *Cav1* and *Cav2* according to the manufacturer's instructions (#100-25636, Bio-Rad Laboratories, Hercules, CA).

### Western blot

CAV1 protein was measured by western blot as previously described (Otis et al., 2011). Briefly, cytosolic fractions of jejunum were run on denaturing SDS-PAGE gels with a Mini-Protean system (Bio-Rad Laboratories), transferred to nitrocellulose, blocked with blocking grade blocker (Bio-Rad Laboratories), and probed with antibodies to CAV1 (#610059, BD Transduction Labs, Lexington, KY; 1:5000 dilution), α-tubulin (#T6199, Sigma-Aldrich; 1:10,000 dilution), goat anti-rabbit IgG–HRP (#170-6515, Bio-Rad Laboratories; 1:5000 dilution), and goat anti-mouse IgG–HRP (#170-6516, Bio-Rad Laboratories; 1:10,000 dilution). Protein expression was imaged by chemiluminescence (SuperSignal West Pico Chemiluminescent Substrate, Pierce, Rockford, IL). Data from three western blots, with five WT and five CAV1^IEC-KO^ mice each, were analyzed with ImageJ software (NIH, Bethesda, MD).

### Experimental diets

Upon weaning, mice were fed CD *ad libitum* (#7012, Harlan Teklad, Fredrick, MD). At 10 weeks of age, mice were separated randomly into groups and either continued on CD, or fed a 60% HFD or 10% fat, nutrient-matched LFD (#D12492 and #D12450J respectively, Research Diets, New Bruswick, NJ) *ad libitum*. Mice fed HFD and LFD were weighed weekly until euthanasia at 26 weeks; *n*=10-15 mice per diet.

### Plasma cholesterol and lipid analysis

Mice were euthanized by CO_2_ inhalation and blood was collected by cardiac puncture. Serum was collected by centrifugation (15 min, 2000 ***g***, 4°C) after allowing blood to clot on ice. Plasma was collected by centrifugation (15 min, 2000 ***g***, 4°C) in EDTA-treated tubes (Sarstedt, Numbrecht, Germany). All serum and plasma samples were snap frozen and stored at −80°C. Kits were used to measure total cholesterol (Infinity Total Cholesterol, Fisher Scientific), free cholesterol (Free Cholesterol E, Wako Diagnostics, Richmond, VA), HDL cholesterol (HDL-C E, Wako Diagnostics), LDL cholesterol (LDL-C Reagent L-type, Wako Diagnostics), NEFAs [HR Series NEFA-HR(2), Wako Diagnostics], and TGs (Infinity Triglycerides, Fisher Scientific) according to the manufacturer's directions. For HFD and LFD male mice, *n*=5-8; for HFD and LFD female mice, *n*=8-9; and for CD male mice, *n*=6-8.

### High-performance liquid chromatography

HPLC-CAD was implemented to measure levels of specific plasma lipids. Lipids were extracted from plasma (50 μl; *n*=6), liver (10-50 mg; *n*=5), and white adipose tissue (15-40 mg; *n*=5) using a modified Bligh–Dyer procedure (Carten et al., 2011), dried under vacuum, and resuspended in the injection solvent HPLC-grade 2-propanol. The investigator that extracted the lipids blinded the samples from the investigator who completed the HPLC experimentation and analysis.

HPLC-CAD was performed using a LPQ-3400RS quaternary pump, WPS-3000TRS autosampler, TCC-3000RS column oven, Accucore C18 column (150×3.0 mm, 2.1 μm particle size) and Dionex Corona Veo charged aerosol detector (all from Thermo Scientific). Lipids were separated over 80 min in a multistep gradient as follows: 0-5 min at 0.8 ml/min in 98.0% mobile phase A (methanol:water:acetic acid, 750:250:4) and 2.0% mobile phase B (acetonitrile:acetic acid, 1000:4); 5-35 min at 0.8-1.0 ml/min in 30-98% A, 2-65% B and 0-5% mobile phase C (2-propanol); 35-45 min at 1.0 ml/min in 0-30% A, 65-95% B and 5% C; 45-73 min at 1.0 ml/min in 60-95% B and 5-40% C; and 73-80 min at 1.0 ml/min in 60% B and 40% C (adapted from personal correspondence with Marc Plante, Thermo Scientific). Following the analytical portion of the gradient, the column was washed for 20 min with 100% mobile phase C at 0.4 ml/min, then returned to 98% A and 2% B at 0.8 ml/min for re-equilibration. Injection volume was between 5 and 25 μl and was adjusted for each sample to produce optimum peak shape for quantitation. The autosampler tray was maintained at 20.0°C and the column oven temperature at 40.0°C.

Chromatographic peaks were identified by comparison with standards (Sigma-Aldrich). Early-eluting peaks not identified as FAs were confirmed as polar lipids by acetone precipitation (adapted from Morris, 1972), and based on their retention times when compared with those of polar lipid standards, were determined to most likely be lysophospholipids (data not shown). Quantitative comparison of the relative amounts of lipid species of interest was performed blinded using Chromeleon 7.2 (Dionex, Sunnyvale, CA). Peak baselines were drawn manually and areas [in picoamperes (pA) min] were determined automatically. For plasma samples, all peak areas were divided by the area of the second lysophospholipid peak in the same sample. This lipid was chosen as it varied the least among all of the samples after accounting for injection volume. For comparison between samples, lipid amounts are expressed as (peak area)/(area of peak 2), both measured in pA×min. For liver and white adipose samples, all peak areas were normalized to percent of total signal to control for unequal extraction efficiencies and differences in starting tissue mass between samples.

### Glucose metabolism

Blood glucose was measured in mice fasted 4 h by tail bleed using a glucometer (AlphaTRAK glucose meter, VWR, Radnor, PA) just prior to euthanasia (*n*=6-10). For intraperitoneal (IP) glucose tolerance test (GTT), mice fasted 4 h were IP-administered 2 g/kg sterilized glucose in water, and blood glucose was measured from the tail vein by glucometer at 0, 15, 30, 60 and 120 min (*n*=9-14). For oral GTT, the same dose of sterile glucose was administered by gavage, and blood glucose was monitored as described (*n*=7-9). Insulin tolerance test (ITT) was performed by administering mice IP insulin (#12585-014, Gibco, Gaithersburg, MD) at a dose of 0.76 U/kg for LFD mice and 1 U/kg for HFD mice; blood glucose was measured as described for GTT (*n*=7-13). GTT and ITT results were analyzed by two-way, and oral GTT by one-way repeated measures ANOVA followed by Tukey's post hoc test.

### Lipid tolerance test

Male and female CAV1^IEC-KO^ and WT littermates were housed on CD until 12 weeks old, fasted 16 h, and gavaged with 10 μl/g body mass 20% emulsified Intralipid (soybean oil) (Sigma). Serum was collected from tail bleed at 0, 1, 2, 3 and 4 h post gavage. TGs and NEFAs were assayed as described in ‘Plasma cholesterol and lipid analysis’ above.

### Statistics

Differences in Cav1-eGFP fluorescence intensities were compared with a one-way ANOVA followed by Tukey's post hoc test. Student's *t*-test was used to compare endocytic cargo membrane fluorescence and RT-PCR measurements of jejunal mRNA. Western blot data from each of three blots were adjusted by expression of the loading control and expressed relative to WT levels, averaged together, and compared by Student's *t*-test. Differences in body mass were compared by linear regression. Plasma lipids identified by HPLC were compared by two-way ANOVA followed by Tukey's post hoc test. Liver and white adipose lipids measured by HPLC were compared by Student's *t*-test. All data analyzed by ANOVA were first confirmed for homogeneity of variance by Bartlett's or Brown–Forsythe tests. Statistics were performed with Prism software (GraphPad, La Jolla, CA).
